# Bitter gourd bioactive peptide alleviates neuronal ferroptosis after spinal cord ischemia-reperfusion injury, combined with emerging cell and animal models

**DOI:** 10.3389/fnut.2026.1850363

**Published:** 2026-05-11

**Authors:** Qiyang Diao, Ming Nuo, Qimuge Suyila, Yongzhen Nie, Xiulan Su

**Affiliations:** 1 Department of Anesthesiology, Peking University Cancer Hospital (Inner Mongolia Campus)/Affiliated Cancer Hospital of Inner Mongolia Medical University, Inner Mongolia Medical University, Hohhot, China; 2 Department of Anesthesiology, Inner Mongolia Autonomous Region People’s Hospital, Hohhot, China; 3 Clinical Medical Research Center of the Affiliated Hospital, Inner Mongolia Medical University, Hohhot, China; 4 Clinical Medical Research Center of Affiliated Hospital, Inner Mongolia Medical University, Inner Mongolia Bioactive Peptide Engineering Laboratory, Hohhot, China

**Keywords:** bitter gourd bioactive peptide, ferroptosis, Nrf2/HO-1, oxidative stress, SCIRI

## Abstract

**Background:**

Spinal cord ischemia-reperfusion injury (SCIRI) remains a major clinical challenge with few effective treatments. Ferroptosis, an iron-dependent form of cell death driven by lipid peroxidation, plays a key role in SCIRI pathology, and activation of the Nrf2/HO-1 pathway can counteract this process. Bitter gourd bioactive peptide (BGBP) is a natural low-molecular-weight peptide with antioxidant properties, but its effect on SCIRI induced ferroptosis is unknown. This study aimed to determine whether BGBP protects against SCIRI by inhibiting neuronal ferroptosis via the Nrf2/HO-1 pathway, using two emerging cell and animal models.

**Methods:**

An *in vitro* chemical hypoxia model was established in BV-2 microglial cells using CoCl_2_, which mimics hypoxic injury without the need for specialized chambers. An *in vivo* rat SCIRI model was created by transient abdominal aortic clamping, a reproducible method that preserves partial spinal cord blood supply. BGBP was applied at its optimal concentration (1.6 mg/mL *in vitro*; 50 mg/kg orally *in vivo*). We assessed cell viability, oxidative stress markers (ROS, MDA, SOD), ferroptosis indicators (Fe^2+^, GSH, GPX4), apoptosis-related proteins (Bcl-2, Bax, Cleaved-Caspase-3), and Nrf2/HO-1 pathway activation by qPCR and western blot. Motor function was evaluated using Tarlov and BBB scores, and spinal cord histopathology was examined by H&E and Nissl staining.

**Results and conclusion:**

BGBP significantly improved BV-2 cell viability under CoCl_2_ induced hypoxia and reduced ROS, MDA, and Fe^2+^ levels while restoring SOD, GSH, and GPX4 activities. It also rebalanced the Bcl-2/Bax ratio and suppressed Cleaved-Caspase-3. Both mRNA and protein levels of Nrf2 and HO-1 were upregulated by BGBP. In the rat SCIRI model, BGBP treatment improved hindlimb motor scores, preserved motor neuron morphology, and reduced histopathological damage, consistent with the *in vitro* findings. BGBP attenuates neuronal ferroptosis and oxidative stress after SCIRI by activating the Nrf2/HO-1 pathway. The combination of the CoCl_2_ induced BV-2 cell model and the rat abdominal aortic clamping model offers a robust and practical methodological platform for studying ferroptosis-targeted neuroprotection.

## 1 Introduction

Spinal cord ischemia-reperfusion injury (SCIRI) is a devastating complication of aortic surgery, with high disability and mortality rates driven by secondary neuronal damage after blood flow restoration (1). Over the years, researchers have developed various experimental models to dissect SCIRI mechanisms. *In vitro*, oxygen-glucose deprivation (OGD) in neuronal cell lines is widely used, but it requires specialized hypoxic chambers and is prone to variability (2). *In vivo*, rodent models of aortic cross-clamping or balloon occlusion have been established, yet some techniques are technically demanding or produce inconsistent injury severity (3). In the present study, we adopted two emerging yet practical model systems. For *in vitro* experiments, we used CoCl_2_ induced chemical hypoxia in BV-2 microglial cells. CoCl_2_ stabilizes hypoxia-inducible factor (HIF) and reproduces key features of hypoxic injury without the need for anaerobic chambers, offering better reproducibility, lower cost, and easier handling than conventional OGD (2, 4). For *in vivo* studies, we employed the rat abdominal aortic clamping model, a well-validated approach that induces transient ischemia of the lower spinal cord by clamping the aorta just below the left renal artery. This model preserves partial blood supply via the anterior spinal artery (5), closely mimicking the clinical scenario of spinal cord ischemia during aortic surgery and allowing consistent assessment of motor function and histopathology. However the mainstream aortic cross-clamping time currently is 30 or 60 min (6). Unlike previous modeling methods, we adopted a 90-min ischemia period and confirmed the success of the modeling through subsequent tests. Compared with 30- and 60-min ischemia periods, our model is more stable. In preliminary dose-finding studies, we compared 30, 60, and 90 min ischemia durations and found that the 90 min protocol produced more consistent histopathological injury and motor deficit scores across animals, with reduced inter-individual variability. Therefore, this duration was adopted for the formal experiments (7). Together, these complementary models provide a robust methodological framework to investigate the molecular mechanisms of SCIRI and evaluate potential therapeutics. The pathological cascade of SCIRI involves oxidative stress and ferroptosis—an iron-dependent, lipid-peroxidation-driven cell death pathway (1). The nuclear factor erythroid 2-related factor 2 (Nrf2)/heme oxygenase-1 (HO-1) signaling axis is a central defense against these processes; its activation enhances antioxidant enzymes, upregulates glutathione peroxidase 4 (GPX4), and maintains iron homeostasis, thereby inhibiting ferroptosis (8). Recent evidence shows that pharmacological activation of Nrf2/HO-1 confers neuroprotection in spinal cord injury models by suppressing neuronal ferroptosis (9), highlighting its therapeutic promise in SCIRI.

Bioactive peptides derived from natural food sources have garnered increasing attention as safe and effective antioxidant agents (10, 11). Bitter gourd, a traditional medicinal and edible plant, contains various bioactive constituents including peptides, polysaccharides, and saponins with documented antioxidant, anti-inflammatory, and neuroprotective properties (12–14). Specifically, bitter gourd extracts have been shown to mitigate oxidative stress in neuronal tissues and improve functional outcomes in models of peripheral neuropathy and cerebral ischemia (15–17). The bitter gourd bioactive peptide (BGBP) used in this study is a low-molecular-weight peptide fraction (< 10 kDa) obtained through enzymatic hydrolysis and ultrafiltration, processes that have been shown to enrich antioxidant peptide components (18). Importantly, emerging evidence suggests that food-derived peptides can chelate free iron ions, scavenge reactive oxygen species (ROS), and modulate the Keap1-Nrf2-HO-1 signaling pathway to protect against oxidative damage (11, 19, 20). However, despite these promising properties, the specific effects of BGBP on spinal cord ischemia-reperfusion injury, particularly its potential to attenuate neuronal ferroptosis via Nrf2/HO-1 activation, have not been systematically investigated.

The present study was therefore designed to address this knowledge gap by exploring whether BGBP exerts neuroprotective effects against SCIRI through inhibition of ferroptosis and oxidative stress via the Nrf2/HO-1 pathway. By integrating *in vitro* CoCl_2_-induced hypoxic injury in BV-2 microglial cells and *in vivo* rat SCIRI models, we aimed to evaluate the therapeutic potential of BGBP in ameliorating neurological deficits and histopathological damage at first; Secondly, elucidate the underlying mechanisms focusing on ferroptosis regulation and Nrf2/HO-1 pathway activation; and provide experimental evidence supporting BGBP as a novel natural therapeutic candidate for SCIRI management at last. This research not only extends the traditional application of bitter gourd into modern neuroprotective strategies but also offers new insights into targeting ferroptosis for spinal cord ischemia-reperfusion injury treatment.

## 2 Materials and methods

### 2.1 Animals and reagents

The research protocol was approved by the Biomedical Ethics Committee of Inner Mongolia Medical University (Approval No.: YKD20252014). Six- to eight-week-old Sprague-Dawley (SD) rats weighing approximately 250 g were obtained from Beijing Sibeifu Biological Co., Ltd. The rats were housed under standard conditions with a 12-h light/dark cycle, maintained at 22–23°C and 50–60% humidity. The animals had *ad libitum* access to food and water. Bitter gourd bioactive peptides (BGBP) were derived from the entire bitter gourd plant through grinding, enzymatic hydrolysis, and ultrafiltration. The resulting BGBP preparation is a low-molecular-weight peptide fraction (< 10 kDa) with a peptide content of approximately 75% (w/w), as determined by the bicinchoninic acid (BCA) method. This preparation method has led to a national invention patent (Patent No. CN116813700A; see Reference list for full patent details) (21). CoCl_2_ was purchased from Merck KGaA (Darmstadt, Germany).

### 2.2 Cell culture and cell model

The BV-2 glial cell line was obtained from the Procell. BV-2 cells were cultured in a 37°C, 5% CO_2_ incubator using BV-2-specific cell culture medium (CM-0493A, Procell, China). CoCl_2_ was used to simulate chemically induced hypoxia in BV-2 cells (22, 23). Cells were seeded into culture dishes and allowed to grow for 24 h. Leveraging the high water solubility of CoCl_2_, solutions of varying CoCl_2_ concentrations were prepared by diluting it in serum-free medium. Cells were then exposed to CoCl_2_ concentrations ranging from 10 to 120 μM for 24 h. Cell viability was assessed using the MTT assay. To account for potential direct interference of cobalt ions with the MTT reagent, cell-free blank controls containing corresponding concentrations of CoCl_2_ were included in each assay, and blank absorbance values were subtracted during data analysis. The concentration that moderately inhibited cell viability was selected for subsequent experiments. Following CoCl_2_ induction of hypoxic injury, cells were treated with varying concentrations of BGBP. The optimal BGBP concentration for subsequent experiments was determined by measuring cell survival rates using the MTT assay. Cells were then divided into four groups: Control (Ctrl), CoCl_2_, Ctrl + BGBP, and CoCl_2_ + BGBP. After treating each group with the optimal BGBP concentration for 24 h, cells were observed under a microscope.

### 2.3 Cell incucyte

After counting BV-2 cells, they were seeded at a density of 5 × 10^3^ cells per well into a 96-well plate, with a final volume of 200 μL. Seeded 96-well plates were placed in a cell culture incubator for 24 h. After 24 h of incubation, drug treatment was administered. Groups included Ctrl, CoCl_2_, Ctrl + BGBP, and CoCl_2_ + BGBP. Each experiment included at least three replicate wells. The treated 96-well plates were loaded into a cell incubator (Essen, United States). Images were captured every 4 h, and data were collected and analyzed after 24 h. Real-time live-cell videos (24 h) are provided as Supplementary Videos 5–8.

### 2.4 Cell ROS detection

According to the manufacturer’s instructions, the ROS level in cells was detected using the oxidation-sensitive fluorescent probe DCFH-DA assay kit (E-BC-K138-F, Elabscience, China). Fluorescence intensity was measured by flow cytometry, and statistical analysis was performed using FlowJo software.

### 2.5 Animal models

**FIGURE 1:**
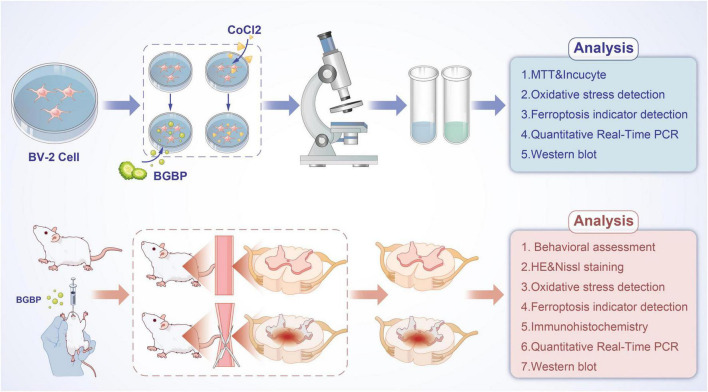
Basic experimental flowchart.

The rats were randomly divided into four groups (*n* = 6) according to the experimental protocol: sham group (Sham), sham-operated group treated with BGBP (Sham + BGBP), model group (Model), and treatment group treated with BGBP (Model + BGBP). The treatment group received prophylactic oral administration of BGBP at a dose of 50 mg/kg/day for 21 days prior to surgery. BGBP administration was continued once daily until euthanasia at 24 h post-reperfusion. The dose of 50 mg/kg/day was selected based on: (1) dose conversion from the optimal *in vitro* concentration (1.6 mg/mL) using the body surface area-based method (24), accounting for typical oral bioavailability of small peptides; and (2) previous literature reporting neuroprotective effects of bitter gourd extracts and bioactive peptides at 25–100 mg/kg in rodents (10–12, 15). (3) The team’s early-stage works (25–27). The Model and Treatment groups underwent rat spinal cord ischemia-reperfusion injury modeling via abdominal aortic clamping (28) as follows: induction with 2% isoflurane inhalation anesthesia. A subcutaneous injection of heparin (2–3 mg/kg) was administered 5 min prior to surgery. A cut was made in the middle of the abdomen, and tissues were dissected layer-by-layer to uncover the abdominal aorta and left renal artery. The abdominal aorta at 0.5 cm below the left renal artery for 90 min to induce ischemia, and then the clamp was released for reperfusion (Figure 1). The sham surgery and treatment groups underwent identical surgical procedures without clamping. The abdominal cavity was closed by saline irrigation. Postoperative care included routine nursing, assisted urination, and ketorolac tromethamine administration as an analgesic. All rats in the surgical groups were observed for 24 h before euthanasia under deep anesthesia with 5% isoflurane for tissue collection. Spinal cord tissue from T10-L5 was harvested for subsequent use. The specific operation is as follows: Twenty-four hours after surgery, rats were euthanized for tissue collection. Each animal was first deeply anesthetized with 5% isoflurane delivered through an induction chamber. Loss of consciousness was confirmed by the absence of the pedal withdrawal reflex. The rat was then placed in dorsal recumbency, and a midline laparotomy was performed to expose the abdominal aorta. Exsanguination was achieved by puncturing the abdominal aorta with a 21-gauge needle. Cessation of heartbeat and respiration was verified before proceeding with spinal cord harvest. This two-step method is consistent with the AVMA Guidelines for the Euthanasia of Animals (2020 Edition). All animal procedures were approved by the Institutional Animal Care and Use Committee of the Biomedical Ethics Committee of Inner Mongolia Medical University (Approval No.: YKD20252014).

### 2.6 Behavioral assessment

The motor function of the hind limbs of rats was assessed using the Tarlov and BBB scales. The Tarlov scale ranged from 0 (no ankle movement) to 4 (normal), whereas the BBB scale ranged from 0 (complete paralysis) to 21 (normal). Observations were conducted at 6, 12, and 24 h postoperatively, with scoring performed independently by two double-blinded evaluators. The scores reported represent the mean of the two evaluators’ scores. The inclined plane test and open-field test were video-recorded; representative videos are shown in Supplementary Videos 1–4.

### 2.7 HE staining

The spinal cord tissue was fixed in 4% paraformaldehyde for 24 h, followed by dehydration and paraffin embedding. Sections 5 μm thick were then cut and deparaffinized. Sections were stained with hematoxylin for 5 min and eosin for 3 min, and then dehydrated with ethanol and xylene. After mounting with neutral resin, the sections were observed under an optical microscope.

### 2.8 Nissl staining

Neuronal damage in the spinal cord tissue was evaluated using Nissl staining as previously described. Paraffin-embedded spinal cord tissue sections (5 μm thick) were stained with tar violet at 56°C for 10 min and differentiated in 1% hydrochloric acid alcohol for several seconds. Sections were dehydrated using ethanol and xylene, mounted with neutral resin, and observed under a light microscope.

### 2.9 Oxidative stress factor assay

The spinal cord tissue was weighed and homogenized in PBS at a ratio of 1:9. For each group, take 10^6^ BV-2 cells and add 300–500 μL of PBS (0.01M, pH 7.4) for homogenization. Centrifuge at 10,000 × g for 10 min at 4°C, then collect the supernatant from the homogenate and determine the concentration of protein with a BCA protein assay kit (E-BC-K318-M, Elabscience, China). The levels of SOD (E-BC-K020-M, Elabscience, China) and MDA (E-BC-K025-M, E-BC-K028-M (cell only), Elabscience, China) in tissues and cells were measured using the corresponding commercial kits, following the manufacturer’s instructions.

### 2.10 ROS detection

Frozen sections were prepared from fresh samples. Then, a 4% paraformaldehyde solution was added on ice for 15 min. After rinsing, the samples were incubated with blocking solution (5% goat serum) at 37°C for 60 min. The cells were then incubated with the oxidation-sensitive fluorescent probe DCFH-DA (1:500 dilution) at 37°C for 60 min. After washing, the samples were incubated with DAPI in the dark for 2 min for nuclear staining. Images were acquired using a laser confocal microscope.

### 2.11 Ferroptosis factor assay

According to the manufacturer’s instructions, the ferrous ion detection kit (E-BC-K773-M, Elabscience, China) was used to measure ferrous ion levels in spinal cord tissue. Following the commercial kit (E-BC-F101, Elabscience, China) protocol, cells were incubated with the ferrous ion-binding fluorescent probe FerroOrange (37°C, 30 min) to detect intracellular ferrous ion levels. Fluorescence intensity was analyzed by flow cytometry and statistically evaluated using the FlowJo software. The GSH Detection Kit (E-BC-K030-M, Elabscience, China) and GPX4 Detection Kit (E-BC-K883-M, Elabscience, China) were used to assess GSH and GPX4 levels in spinal cord tissue and cells.

### 2.12 Quantitative real-time PCR (qPCR) assay

Total RNA was extracted from the spinal cord tissue and cells using the TRIzol Kit (ET111-01-V2, TransGen, China) following the manufacturer’s protocol. A PrimeScript RT-PCR kit (A5001; Promega, United States) was used for first-strand cDNA synthesis. Real-time qPCR was performed using SYBR Premix Ex Taq (A6001; Promega, United States) to detect the expression of apoptosis-related proteins Bcl-2, Bax, and Caspase-3, as well as the ferroptosis-associated proteins Nrf2 and HO-1. All reactions were conducted in triplicates. Primers specific for Bcl-2, Bax, Caspase-3, Nrf2, and HO-1 were purchased from Sangon Biotechnology (Shanghai, China). All genes were quantified using the 2^–ΔΔCT^ formula with GAPDH as the internal control. The primer sequences used in this study are listed in Table 1. For the *in vitro* experiments using mouse BV-2 cells, the primer sequences listed in Table 2 were used.

**TABLE 1:** qPCR primers (rat).

Gene	Forward sequence	Reverse sequence
Bcl-2	TGGATGACTGAGTACCTGAACCG	CAGCCAGGAGAAATCAAACAGAGG
Bax	CACCTGAGCTGACCTTGGAG	TCCTCTGCAGCTCCATGTTG
Caspase-3	AGAGCTGGACTGCGGTATTGAG	GCGGTAGAGTAAGCATACAGGAAG
Nrf2	CTACAGATGCCAACCACTGAAAGG	TCCCAACACAGGTACATAAGAATGAAG
HO-1	GAACTTTCAGAAGGGTCAGGTGTC	CTGCTTGTTTCGCTCTATCTCCTC
GAPDH	TGCCACTCAGAAGACTGTGG	TTCAGCTCTGGGATGACCTT

**TABLE 2:** qPCR primers (mouse).

Gene	Forward sequence	Reverse sequence
Bcl-2	CGGGAGATCGTGATGAAGTACATAC	TCAGGCTGGAAGGAGAAGATGC
Bax	CAGGTGATTGAGTTGGAGAGGAAG	ACTTGGGTTTCGGTGAGTTTGAG
Caspase-3	GCTGGACTGTGGCATTGAGAC	AGGAATAGTAACCAGGTGCTGTAGAG
Nrf2	GCCACCGCCAGGACTACAG	TGCTCAGAAACCTCCTTCCAAAAC
HO-1	AGACCGCCTTCCTGCTCAAC	GACGAAGTGACGCCATCTGTG
GAPDH	ACGGCAAATTCAACGGCACAG	ACACCAGTAGCATCCACGACATAC

### 2.13 Immunohistochemistry

The tissue sections were dewaxed and placed in citric acid repair solution (pH: 6.0) for 10 min of heat repair at 95°C to expose the antigen. After cooling to 24°C, they were inactivated with 3% H_2_O_2_ solution at 37°C for 30 min, blocked with 10% goat serum, and then incubated with primary antibodies against Nrf2 (16396-1-AP, Proteintech, 1:400, China), HO-1 (10701-1-AP, Proteintech, 1:800, China), Bax (AF0120, Affinity, 1:800, China), Bcl-2 (AF6139, Affinity, 1:800, China), and Cleaved-Caspase-3 (AF7022, Affinity, 1:800, China) (4°C, overnight). The next day, the sections were washed with PBS and then incubated with secondary antibodies (KIT-5010, Maxim, China) (37°C, 30 min). Thereafter, the samples were treated with DAB chromogenic solution. After chromogenesis, sections were counterstained with hematoxylin, dehydrated, and mounted. Finally, the stained sections were observed under an optical microscope.

### 2.14 Western blotting

**FIGURE 2:**
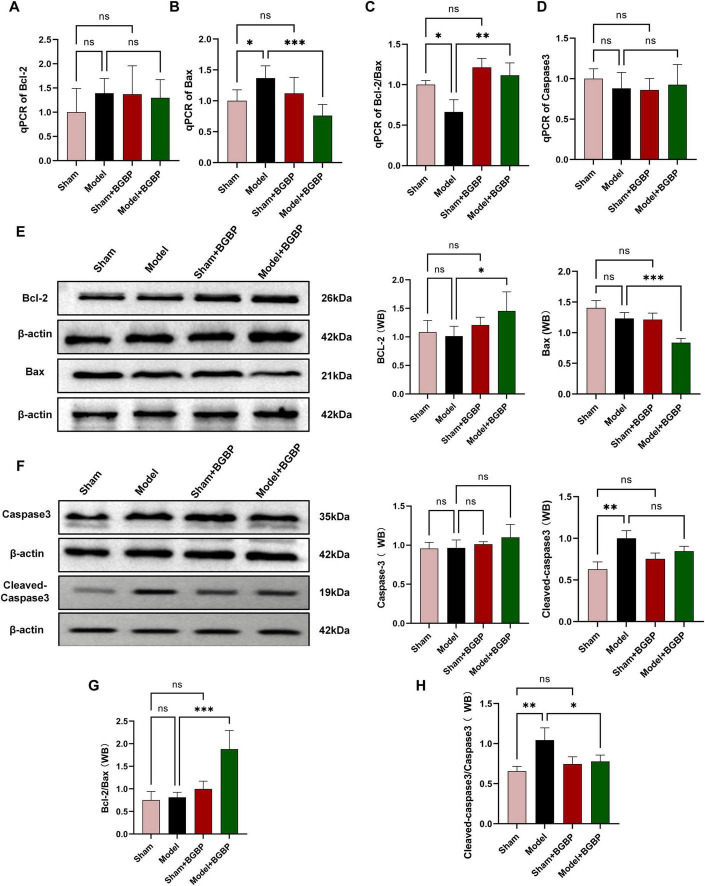
*In vivo* experiments on BGBP regulation of apoptosis. **(A)** Bcl-2 gene expression levels. **(B)** Bax gene expression levels. **(C)** Relative Bcl-2/Bax expression levels (qPCR). **(D)** Caspase-3 gene expression levels. **(E)** Western blot analysis of Bcl-2 and Bax. **(F)** Western blot analysis of Caspase-3 and Cleaved-Caspase-3. **(G)** Relative expression levels of Bcl-2/Bax (Western blot). **(H)** Relative expression levels of Cleaved-Caspase-3/Caspase-3 (Western blot) (*n* = 3). *p < 0.05, **p < 0.01, ***p < 0.001.

Spinal cord tissues and cells were lysed using a Total Protein Extraction Kit (E-BC-E002, Elabscience, China). The protein concentration was determined using a BCA Protein Assay Kit (E-BC-K318-M, Elabscience, China). Equal amounts of proteins were separated by 10% SDS-PAGE and transferred to PVDF membranes (IPVH00010, Millipore, Germany). The membrane was incubated with 5% non-fat milk at room temperature for 90 min, followed by overnight incubation at 4 °C with primary antibodies against Nrf2 (16396-1-AP, Proteintech, 1:2,000, China), HO-1 (10701-1-AP, Proteintech, 1:5,000, China), Bax (AF0120, Affinity, 1:1,000, China), Bcl-2 (AF6139, Affinity, 1:1,000, China), Caspase-3 (9662, CST, 1:1,000, United States; detects both full-length and cleaved forms), and Cleaved-Caspase-3 (68773-1-Ig, Proteintech, 1:5,000, China; specific for the cleaved 17/19 kDa fragment). Antibody dilutions were optimized to clearly distinguish the full-length (35 kDa) and cleaved (17/19 kDa) bands without signal overlap. In Figure 2F, the total Caspase-3 signal shown corresponds to the full-length 35 kDa band. The membrane was then incubated with HRP-labeled secondary antibody (SA00001-2, Proteintech, 1:5,000, China) at room temperature for 90 min, using β-actin (66009-1-Ig, Proteintech, 1:20,000, China) and GAPDH (60004-1-Ig, Proteintech, 1:50,000, China) as controls. Protein bands were visualized using an ECL chemiluminescent kit (BL520A, Biosharp, China), developed with a chemiluminescent detection system, and quantified using the ImageJ software.

### 2.15 Statistical analysis

Data are presented as the mean ± standard deviation of at least three independent experiments. Statistical analysis was performed using GraphPad 9.5.0 software (GraphPad Software, La Jolla, United States). The Shapiro-Wilk test was used to assess the normal distribution of data in each group. Data that were normally distributed and had equal variances were analyzed using one-way ANOVA, and pairwise comparisons between groups were conducted using the LSD test. Data that did not meet the normal distribution or had unequal variances were analyzed using the rank sum test. *p* < 0.05 was considered statistically significant.

## 3 Results

### 3.1 Bitter gourd bioactive peptides alleviate hypoxia-induced cellular damage and determine their optimal therapeutic concentration

**FIGURE 3:**
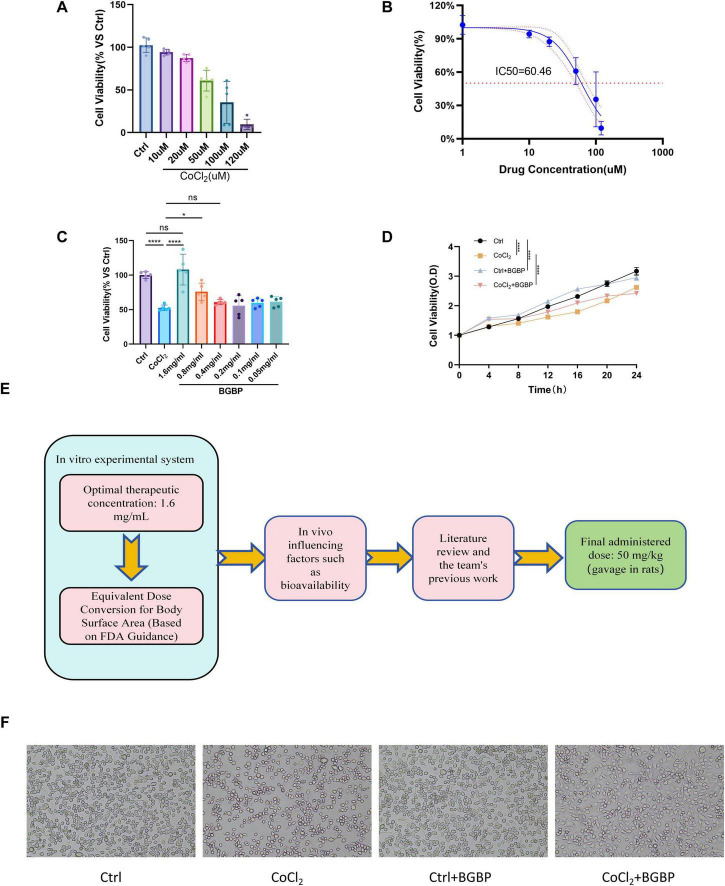
Optimal therapeutic concentration of bitter gourd bioactive peptides and their protective effects on cell viability. **(A,B)** Determination of CoCl_2_ IC_50_ for BV-2 cells using the MTT assay. **(C)** Determination of optimal therapeutic concentration of BGBP using the MTT assay. **(D)** Real-time monitoring of cell growth curves using the Incucyte system. **(E)** Conversion of *in vitro* optimal concentration to *in vivo* rat dosage. The *in vitro* optimal concentration of 1.6 mg/mL was selected based on MTT assays. The oral rat dose of 50 mg/kg/day was derived considering: (1) body surface area (BSA)-based normalization; (2) the low oral bioavailability characteristic of peptides; and (3) empirical validation from our team’s published studies using this same BGBP preparation at 50 mg/kg/day in rat models. **(F)** Microscopic image of cells cultured for 24 h under optimal conditions (*n* = 5) in **(A–C)**. For **(D)**, *n* = 10 represents 10 replicate wells from a single representative experiment; independent experiments were repeated three times, *n* = 3 in panel **(F)**. Data are presented as mean ± SD. Statistical analysis was performed using one-way ANOVA followed by LSD *post-hoc* test. *p < 0.05, ****p < 0.0001.

To establish a stable *in vitro* model and determine the optimal therapeutic parameters of BGBP, we first conducted an MTT assay. Results indicated that the concentration produced approximately 50% inhibition of cell viability (IC_50_ ≈ 60 μM) was selected for subsequent experiments to ensure a moderate yet consistent hypoxic injury. This concentration range is consistent with previously reported CoCl_2_ treatment parameters for BV-2 microglial cells, where concentrations between 50 and 500 μM have been shown to induce dose-dependent hypoxic responses (22, 29). The 60 μM concentration was validated across three independent experiments with consistent effects on cell viability and oxidative stress markers (Figures 3A,B). When determining the therapeutic concentration of BGBP, we found that at 1.6 mg/mL, the peptides significantly increased the survival rate of CoCl_2_-treated cells (vs. CoCl_2_, *p* < 0.0001) without exhibiting significant toxicity to normal cells. Therefore, this concentration was established as the optimal therapeutic concentration for *in vitro* use (Figure 3C).

After determining the optimal modeling concentration and therapeutic concentration, BV-2 cells were divided into four groups: Ctrl, CoCl_2_, Ctrl + BGBP, and CoCl_2_ + BGBP. Following treatment under the specified conditions, cells were cultured for 24 h and photographed under a microscope. As shown in Figure 3F, compared with the control group, the CoCl_2_ group exhibited a significant reduction in BV-2 cell numbers. By contrast, both CoCl_2_ + BGBP and Ctrl + BGBP groups demonstrated markedly increased BV-2 cell numbers, more intact morphology, and reduced numbers of dead floating cells.

To dynamically evaluate the protective effects of BGBP, we employed the Cell Incucyte system for real-time imaging and cell growth curve analysis for 24 h. As shown in Figure 3D, control (Ctrl) cells exhibited a standard logarithmic growth curve. By contrast, cell growth in the CoCl_2_ group was significantly inhibited (*p* < 0.0001). Compared to the CoCl_2_ group, cell growth in the CoCl_2_ + BGBP group showed marked improvement (*p* < 0.0001), indicating enhanced proliferative activity. To quantify this difference, we calculated the relative cell viability based on 24-h cell activity data. Compared to the Ctrl group, the CoCl_2_ group exhibited cell viability at 4, 8, 12, 16, 20, and 24 h of 105.6 ± 8.1%, 72.7 ± 5.6%, 64.1 ± 5.2%, 67.1 ± 5%, and 75 ± 5.4%, respectively. The Ctrl + BGBP group exhibited cell viability at 4, 8, 12, 16, 20, and 24 h equivalent to 204.2 ± 16.8%, 122.4 ± 6.6%, 118.6 ± 6.5%, 119.3 ± 8.2%, 99.6 ± 7.5%, and 90.1 ± 6.7% of the Ctrl group, respectively; The cell viability of the CoCl_2_ + BGBP group at 4, 8, 12, 16, 20, and 24 h was 190.5 ± 20.7%, 100 ± 7.2%, 81.1 ± 4.8%, 83.4 ± 4.6%, 76.4 ± 4.8%, and 65.5 ± 3.9% of the Ctrl group, respectively. Notably, normal cells treated with BGBP (Ctrl + BGBP) exhibited a faster growth rate than the control group during the early culture stages (*p* < 0.0001). By the end of the observation (24 h), the growth rates stabilized, likely due to premature contact inhibition caused by excessive cell proliferation, leading to a plateau phase.

Based on the optimal *in vitro* concentration (1.6 mg/mL), we calculated the equivalent dose for rats to be approximately 50 mg/kg using the body surface area-based equivalent dosage method (Figure 3E). This dose was subsequently administered by oral gavage *in vivo*.

### 3.2 Bitter gourd bioactive peptides improve neurological recovery and mitigate histopathological damage in SCIRI rats

**FIGURE 4:**
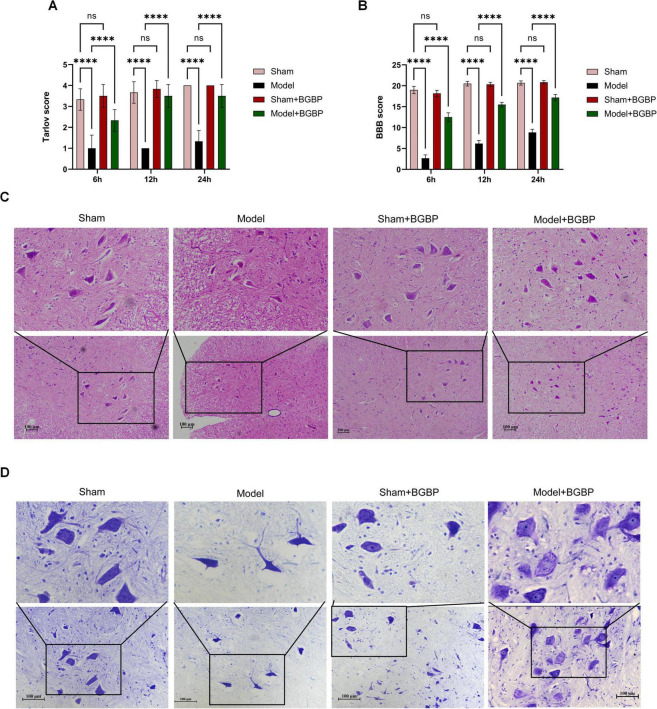
BGBP improves neurological function and tissue morphology in SCIRI rats. **(A)** Tarlov score. **(B)** BBB score. **(C)** HE staining. Low-magnification overview images captured at 10 × objective; insets show enlarged views of the boxed regions captured at 40 × objective. Scale bars: 100 μm (overview), 25 μm (inset). **(D)** Nissl staining. Overview images captured at 10 × objective; insets captured at 40 × objective. Scale bars: 100 μm (overview), 25 μm (inset) (*n* = 3). ****p < 0.0001.

As shown in Figures 4A,B, compared to the sham group, rats in the model group exhibited significantly reduced Tarlov and BBB scores (*p* < 0.0001). Following BGBP treatment (50 mg/kg), the Model + BGBP group demonstrated a significant improvement in neurological function scores compared to the Model group (*p* < 0.0001).

The histological analysis results were highly consistent with behavioral scoring. As shown in Figures 4C,D, HE and Nissl staining revealed a sparse arrangement of motor neurons in the anterior horn of the spinal cord in the model group, with dissolved Nissl bodies and severe cellular damage. By contrast, BGBP treatment significantly improved the neuronal morphology, preserved more Nissl bodies, and reduced neuronal loss. These findings indicate that BGBP alleviates the neurological deficits and histopathological damage induced by SCIRI.

### 3.3 Immunohistochemical observations revealed that bitter gourd bioactive peptides regulate apoptosis in spinal cord tissue and modulate the *in situ* expression of key proteins in the Nrf2/HO-1 pathway

To further investigate the potential mechanisms of BGBP at the level of *in situ* protein expression, we used immunohistochemistry to examine the expression and distribution of key proteins involved in apoptosis and the Nrf2/HO-1 pathway in spinal cord tissues.

**FIGURE 5:**
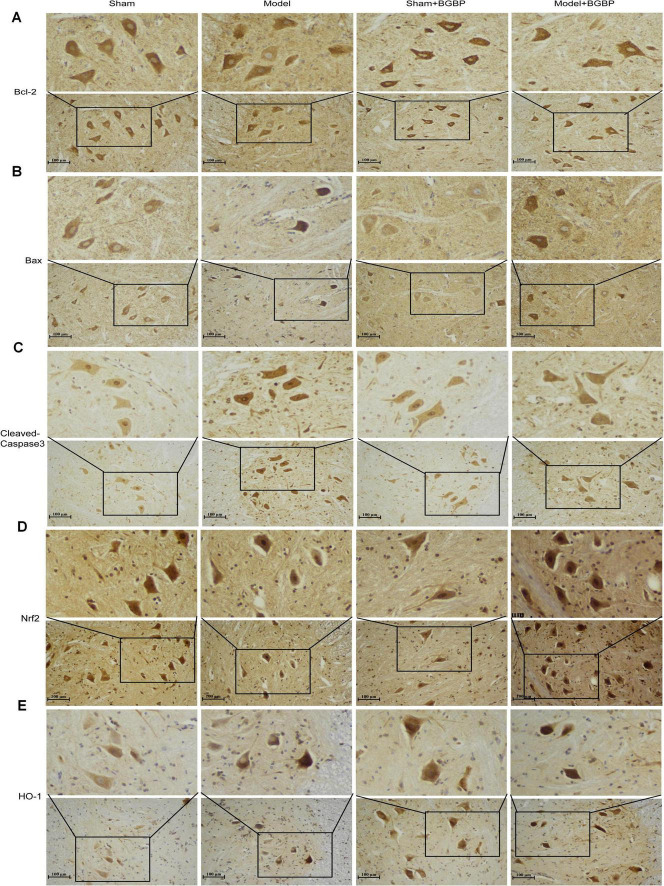
*In situ* expression of key proteins regulated by BGBP in spinal cord tissue. **(A)** Bcl-2, cytoplasm. **(B)** Bax, cytoplasm. **(C)** Cleaved-Caspase-3, cytoplasm. **(D)** Nrf2, cytoplasm, nucleus. **(E)** HO-1, cytoplasm. All immunohistochemistry images were captured at 20 × objective; insets show enlarged views of the boxed regions captured at 40 × objective. Scale bars: 50 μm (main), 25 μm (inset) (*n* = 3).

Microscopic observation revealed that compared with the sham group, the model group exhibited enhanced immunoreactivity for the pro-apoptotic protein Bax in the motor neurons of the spinal cord anterior horn (Figure 5B). By contrast, the immunoreactive signal for the anti-apoptotic protein Bcl-2 showed a decreasing trend (Figure 5A). Crucially, the activated form of Cleaved-Caspase-3 exhibited distinct positive signals in neurons of the model group (Figure 5C), indicating activated apoptosis. Following BGBP treatment, the expression of these proteins exhibited reversible changes: positive signals for Bax and Cleaved-Caspase-3 markedly diminished, while Bcl-2 positive reactions partially recovered (Figures 5A–C).

Simultaneously, our observation of proteins in the Nrf2/HO-1 pathway revealed that, compared to the model group, Nrf2-positive signals were distributed in both the cytoplasm and nucleus, consistent with its transcription factor function. Notably, BGBP-treated sections showed enhanced nuclear Nrf2 immunoreactivity compared with the model group, suggesting increased Nrf2 nuclear translocation upon BGBP treatment (Figure 5D). Furthermore, the protein expression of its downstream target molecule HO-1 showed a marked upward trend (Figure 5E). Nrf2-positive signals were distributed in both the cytoplasm and nucleus, consistent with its transcription factor function. HO-1-positive signals were primarily localized to the cytoplasm. These microscopic observations provided crucial *in situ* expression evidence for our subsequent quantitative protein analysis, preliminarily suggesting that the therapeutic effects of BGBP may be associated with tissue-specific regulation of apoptosis and activation of the Nrf2/HO-1 pathway.

### 3.4 Bitter gourd bioactive peptides alleviate oxidative stress damage in *in vitro* and *in vivo* models

**FIGURE 6:**
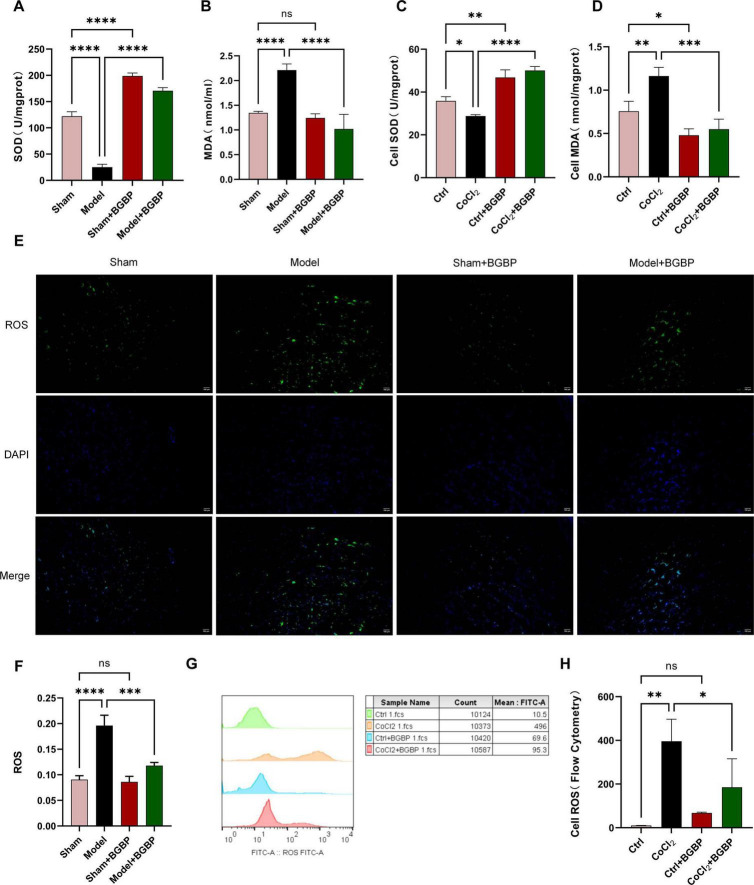
Markers of oxidative stress **(A)** SOD in rat spinal cord tissue. **(B)** MDA in rat spinal cord tissue. **(C)** SOD in BV-2 cells. **(D)** MDA in BV-2 cells. **(E)** Representative fluorescence micrographs of ROS in rat spinal cord tissue. **(F)** Quantification of ROS fluorescence intensity in rat spinal cord tissue. **(G)** Flow cytometry detection of ROS in BV-2 cells. **(H)** Levels of ROS in BV-2 cells (*n* = 3). *p < 0.05, **p < 0.01, ***p < 0.001, ****p < 0.0001.

In this study, we investigated the antioxidant effects of BGBP. In the spinal cord tissue of rats with SCIRI, the model group exhibited significantly decreased SOD activity (Figure 6A) compared to the sham group (*p* < 0.0001). By contrast, the MDA content (Figure 6B) and ROS levels (Figures 6E,F) were significantly elevated (*p* < 0.0001). The BGBP treatment reversed these changes (*p* < 0.001), and the treated group showed a marked increase in SOD activity compared with the sham group (*p* < 0.0001). *In vitro*, CoCl_2_ treatment significantly increased MDA (Figure 6D) and ROS (Figures 6G,H) levels in neurons compared to those in the control group (*p* < 0.01). BGBP significantly cleared excess MDA and ROS (*p* < 0.05) and exhibited the same SOD upregulation effect (Figure 6C) as that observed in the *in vivo* experiments (*p* < 0.01). These *in vivo* and *in vitro* results collectively demonstrate that BGBP possesses potent antioxidant capacity.

### 3.5 Bitter gourd bioactive peptides inhibit ferroptosis in *in vitro* and *in vivo* models

**FIGURE 7:**
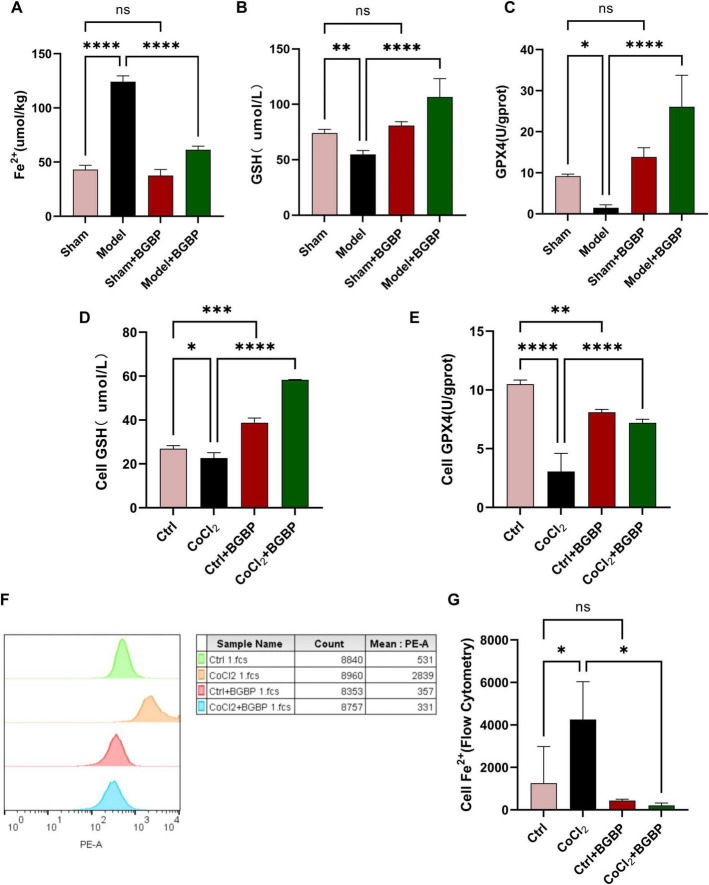
Markers of ferroptosis **(A)** Fe^2+^ levels in rat spinal cord tissue. **(B)** GSH levels in rat spinal cord tissue. **(C)** GPX4 levels in rat spinal cord tissue. **(D)** GSH levels in BV-2 cells. **(E)** GPX4 levels in BV-2 cells. **(F)** Representative flow cytometry histograms of Fe^2+^ levels in BV-2 cells. **(G)** Quantification of Fe^2+^ mean fluorescence intensity (MFI) in BV-2 cells (*n* = 3). *p < 0.05, **p < 0.01, ***p < 0.001, ****p < 0.0001.

Given the pivotal role of ferroptosis in SCIRI, we examined the relevant biomarkers. *In vivo* experiments revealed significant Fe^2+^ accumulation (Figure 7A) in the spinal cord tissue of the model group (vs. sham, *p* < 0.0001) accompanied by markedly reduced GSH levels (Figure 7B) and GPX4 enzyme activity (Figure 7C) (*p* < 0.05). BGBP treatment restored iron homeostasis, increased GSH content, and improved GPX4 activity (*p* < 0.0001). *In vitro* experiments corroborated these findings; BGBP similarly reduced intracellular Fe^2+^ levels (Figures 7F,G) (*p* < 0.05) and increased GSH content (Figure 7D) and GPX4 activity (Figure 7E) (*p* < 0.0001). These data indicated that BGBP is an effective inhibitor of ferroptosis.

### 3.6 Bitter gourd bioactive peptides resist apoptosis by regulating the Bcl-2/Bax balance and inhibiting Caspase-3 activation

To investigate whether BGBP exert their protective effects by inhibiting apoptosis, we examined key markers of the apoptotic pathway at both the gene transcription and protein expression levels.

**FIGURE 8:**
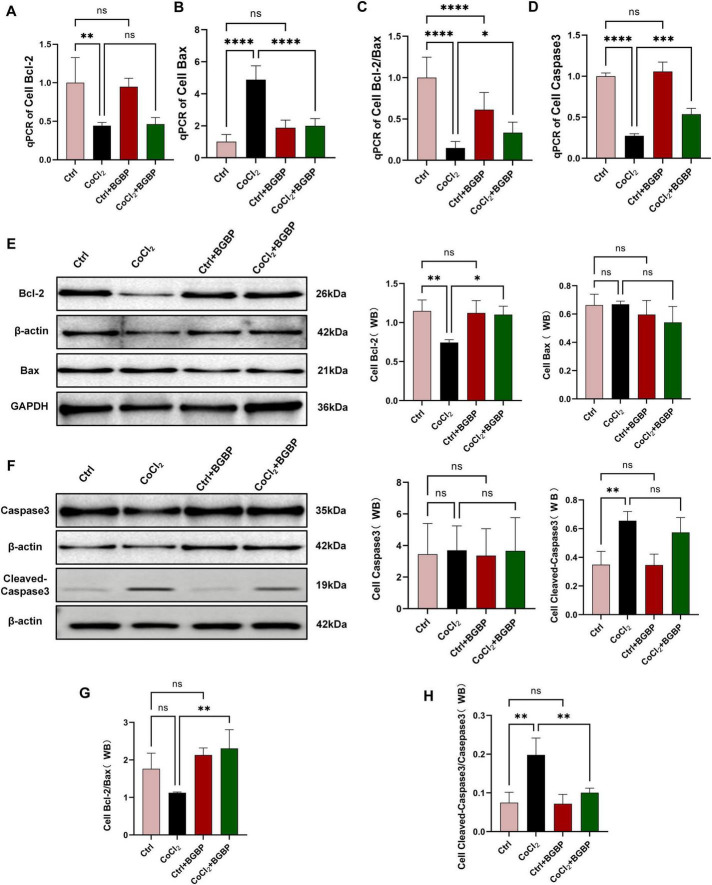
*In vitro* experiments on BGBP regulation of apoptosis. **(A)** Cell Bcl-2 gene expression levels. **(B)** Cell Bax gene expression levels. **(C)** Relative cell Bcl-2/Bax expression levels (qPCR). **(D)** Cell Caspase-3 gene expression levels. **(E)** Western blot of Bcl-2 and Bax. **(F)** Western blot of Caspase-3 and Cleaved-Caspase-3. **(G)** Relative expression levels of Bcl-2/Bax (Western blot). **(H)** Relative expression levels of Cleaved-Caspase-3/Caspase-3 (Western blot) (*n* = 3). *p < 0.05, **p < 0.01, ***p < 0.001, ****p < 0.0001.

At the transcriptional level, qPCR results revealed that the mRNA expression of the proapoptotic gene Bax (Figures 2B, 8B) was significantly upregulated (*p* < 0.05) in both *in vivo* SCIRI and *in vitro* CoCl_2_ injury models. In contrast, the mRNA expression of the anti-apoptotic gene Bcl-2 (Figures 2A, 8A) showed no significant changes. Notably, although the change in expression of Bcl-2 alone did not reach statistical significance in some groups, calculation of the Bcl-2/Bax ratio (Figures 2C, 8C), a more reliable indicator for apoptosis assessment, revealed that this ratio was significantly reduced in both the Model and CoCl_2_ groups (*p* < 0.05). Following BGBP treatment, this ratio was significantly restored (*p* < 0.05). The *In vivo* experiments revealed no significant differences in Caspase-3 mRNA expression (Figure 2D) between groups. However, the *in vitro* experiments revealed significantly reduced Caspase-3 mRNA expression (Figure 8D) in the CoCl_2_ group. This anomaly may have resulted from excessive mRNA degradation due to excessive cell death in the CoCl_2_ group.

Western blot analysis further validated the aforementioned findings at the protein level. As shown in Figure 2, *in vivo* experiments revealed that Bcl-2 protein expression (Figure 2E) exhibited no significant trend, whereas Bax protein expression (Figure 2E) was upregulated, which was consistent with the gene expression trend. BGBP treatment (Figure 2G) reversed this trend by significantly increasing the Bcl-2/Bax ratio (*p* < 0.001).

*In vitro* experiments revealed a significant downregulation of Bcl-2 (Figure 8E) in the CoCl_2_ group (*p* < 0.01), whereas no significant difference was observed in Bax (Figure 8E). BGBP treatment (Figure 8G) significantly increased the Bcl-2/Bax ratio (*p* < 0.01) and inhibited apoptosis.

To clarify the effect of BGBP on the execution phase of apoptosis, we assessed the activation level of Caspase-3 by calculating the ratio of Cleaved-Caspase-3 (Figures 2F, 8F) to total Caspase-3 (Figures 2F, 8F). The results showed that the Cleaved-Caspase-3/Caspase-3 ratio (Figures 2H, 8H) was significantly elevated in both the model group and CoCl_2_ group (*p* < 0.01). BGBP treatment significantly inhibited Caspase-3 activation, resulting in a significantly reduced ratio compared to that in the Model and CoCl_2_ groups (*p* < 0.05).

This multilevel evidence, spanning from genes to proteins and from regulatory to execution points, collectively demonstrates that BGBP exerts an anti-apoptotic effect by modulating the Bcl-2/Bax balance, thereby inhibiting the activation of downstream Caspase-3.

### 3.7 Bitter gourd bioactive peptides activate the Nrf2/HO-1 signaling pathway

**FIGURE 9:**
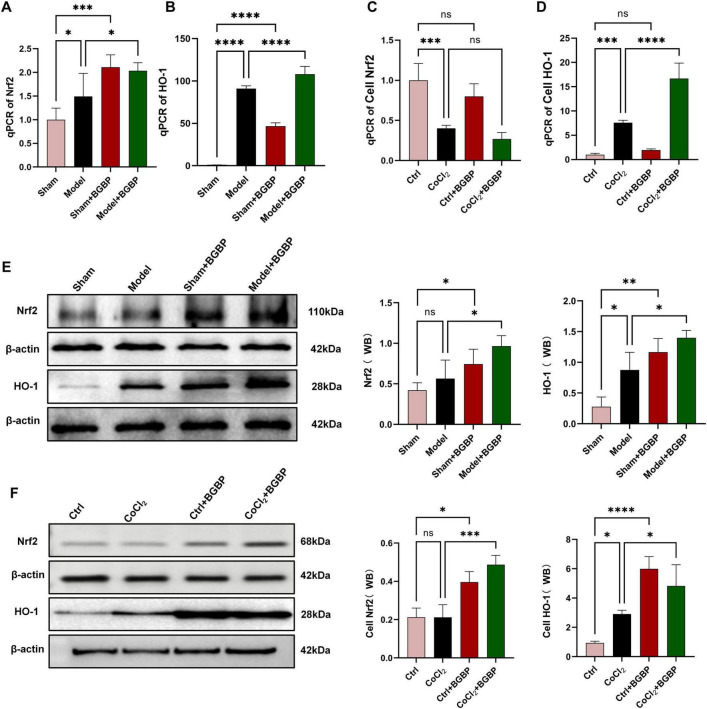
BGBP regulation of the Nrf2/HO-1 pathway. **(A,B)** *In vivo* gene expression levels of Nrf2/HO-1. **(C,D)** *In vitro* gene expression levels of Nrf2/HO-1. **(E)** *In vivo* western blot of Nrf2/HO-1. **(F)** *In vitro* western blot of Nrf2/HO-1 (*n* = 3). *p < 0.05, **p < 0.01, ***p < 0.001, ****p < 0.0001.

Given the central role of the Nrf2/HO-1 pathway in antioxidant defense and antiferroptosis, we assessed its activation status. qPCR (Figures 9A−D) and western blotting (Figures 9E,F) collectively demonstrated that BGBP treatment significantly upregulated the mRNA and protein expression levels of Nrf2 (Figures 9A,E, *p* < 0.05) and its downstream target gene HO-1 (Figures 9B,E, *p* < 0.05) in the spinal cord tissue in the *in vivo* model.

Western blot analysis of *in vitro* cell models confirmed that BGBP significantly enhanced Nrf2/HO-1 protein expression (*p* < 0.05) (Figures 9D,F). Notably, qPCR results indicated no significant changes in Nrf2 mRNA levels (Figure 9C) within the cells. This suggests that BGBP may primarily stabilize the Nrf2 protein through post-translational mechanisms. Integrating the *in vivo* and *in vitro* data, we conclude that activation of the Nrf2/HO-1 pathway represents a key molecular mechanism underlying the neuroprotective effects of BGBP.

## 4 Discussion

Bitter gourd, a common dual-use ingredient in food medicine in China, embodies the dual identity of food and medicine, making it an ideal model for interpreting the concept of “food as medicine.” The extensive history of folk consumption suggests its potential value for improving metabolic health (30). This study does not merely replicate the traditional uses of bitter gourd, but focuses on its highly active extract (BGBP). It aims to reveal the material basis and mechanisms of its therapeutic effects at the molecular level, thereby bridging the traditional experience with modern drug therapy.

**FIGURE 10:**
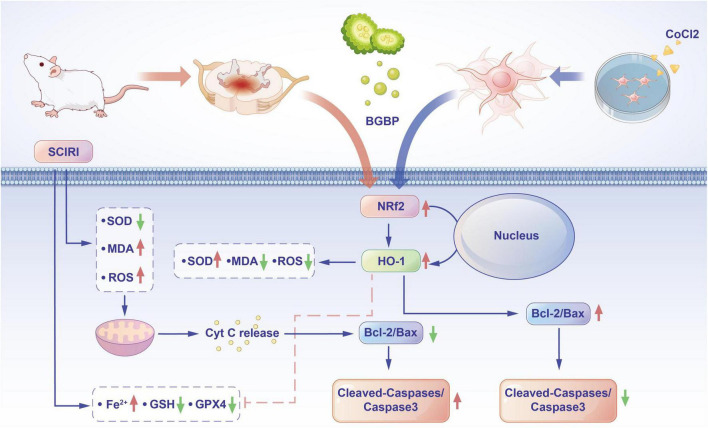
BGBP activates the Nrf2/HO-1 pathway to inhibit neuronal ferroptosis and mitigate spinal cord ischemia-reperfusion injury.

In this study, CoCl_2_ was employed to induce hypoxia-induced injury in BV-2 cells to establish an *in vitro* SCIRI model. Abdominal aortic occlusion was used to establish an *in vivo* rat model of spinal cord ischemia-reperfusion injury. Through *in vivo* and *in vitro* experiments, we investigated the mechanism by which the BGBP regulates ferroptosis in rat spinal cord ischemia-reperfusion injury via the Nrf2/HO-1 pathway (Figure 10). This is the first study to investigate the role and mechanism of BGBP in ferroptosis during spinal cord ischemia-reperfusion injury, further confirming its effects on the Nrf2/HO-1 signaling pathway. Unlike previous studies, we found that the sham group (Sham) also experienced mild oxidative stress damage owing to surgical manipulation and anesthesia. Therefore, a Sham + BGBP group was established for comparison to further clarify the role of BGBP in the regulation of oxidative stress.

During behavioral scoring, we observed that hindlimb motor function in the model group gradually recovered over time, although recovery took longer and scores were lower than those in the Model + BGBP group. No significant paraplegia was observed in any patient. This may be attributed to the dual blood supply to the rat spinal cord provided by the anterior and posterior spinal arteries (31). The abdominal aortic modeling method occludes the posterior spinal artery while leaving the anterior spinal artery intact, thereby maintaining a partial blood supply to the spinal cord. Subsequent observation of motor neuron morphology in the anterior horn of the spinal cord via HE and Nissl staining, along with detection of the oxidative stress markers SOD and MDA, confirmed the success of the modeling procedure.

Our experimental results demonstrated that BGBP intervention significantly enhanced neurological recovery following SCIRI, as confirmed by behavioral assessments of the Tarlov and BBB scores. Concurrently, histological analysis (using HE and Nissl staining) revealed that BGBP mitigated the damage to motor neurons in the anterior horn of the spinal cord. Mechanistically, BGBP comprehensively modulated oxidative stress responses, as evidenced by increased SOD activity, reduced MDA content, and diminished ROS accumulation. Importantly, we found that BGBP restored intracellular iron homeostasis by reducing ferrous ion accumulation, elevating GSH levels, and enhancing GPX4 enzyme activity, collectively indicating the suppression of ferroptosis (32). Molecular biology experiments further confirmed that the protective effects of BGBP were closely associated with activation of the Nrf2/HO-1 signaling pathway.

Secondary injury following SCIRI is primarily driven by oxidative stress (1), Ferroptosis, an iron-dependent form of regulatory cell death, is gaining increasing attention for its role in neurological disorders (33). Our study revealed decreased SOD activity and elevated MDA levels in both Model and CoCl_2_ groups, indicating increased oxidative stress. Concurrently, ferrous ion accumulation, GSH depletion, and reduced GPX4 activity collectively confirmed ferroptosis. This aligns with recent studies on the role of ferroptosis in cerebral ischemia-reperfusion injury (34, 35). BGBP intervention significantly reversed these alterations through three mechanisms: first, by enhancing cellular antioxidant capacity (increased SOD activity); second, by inhibiting lipid peroxidation (reduced MDA levels); and third, by restoring key ferroptosis markers to normal levels (decreased ferrous ions, increased GSH, and GPX4). This multitarget action pattern suggests that BGBP may coordinate these effects by regulating common upstream pathways, with the Nrf2/HO-1 pathway emerging as a potential core regulatory hub.

The Nrf2/HO-1 pathway is a core component of the cellular antioxidant defense system and plays a crucial role in counteracting oxidative stress and inhibiting ferroptosis (36). A key highlight of our research was the revelation of the activating effects of BGBP on this pathway, through a combination of *in vivo* and *in vitro* experiments.

Notably, we observed consistent upregulation of Nrf2 at both the mRNA and protein levels in our *in vivo* experiments, which is consistent with the findings of Hu, who reported that β-caryophyllene inhibits ferroptosis by activating the Nrf2/HO-1 pathway (35). *In vitro* experiments revealed a more complex regulatory mechanism; although BGBP did not significantly alter Nrf2 mRNA levels, it markedly increased its protein expression. This discrepancy between the transcriptional and translational levels suggests that BGBP primarily stabilizes the Nrf2 protein through post-translational regulatory mechanisms. This finding is similar to those of studies showing that Moracin N upregulates Nrf2 expression by blocking the Keap1-Nrf2 interaction (34). Therefore, BGBP may primarily stabilize the Nrf2 protein through post-translational mechanisms, such as interference with the Keap1-Nrf2 interaction or inhibition of Nrf2 ubiquitination and proteasomal degradation. This mechanism is consistent with recent findings on food-derived antioxidant peptides, which have been shown to activate the Nrf2/Keap1 pathway primarily through post-translational regulation (37).

Regardless of the specific mechanism involved, the activation of Nrf2 ultimately leads to increased expression of its downstream target gene, HO-1. Upregulation of HO-1 alleviates intracellular iron overload, a core trigger of ferroptosis, by degrading heme to produce biliverdin, carbon monoxide, and iron ions, thereby activating iron export proteins (38). In our study, the significant upregulation of HO-1 expression in both the Model + BGBP and CoCl_2_ + BGBP groups, along with the subsequent suppression of ferroptosis, fully substantiates the rationale for the protective effects of BGBP via the Nrf2/HO-1 pathway.

The multilevel evidence chain established in this study significantly enhanced the reliability of our conclusions. *In vivo* studies have demonstrated that BGBP improves neurological function and mitigates histological damage. *In vitro* studies have shown that BGBP promotes cell growth and reduces hypoxia-induced injury. Both approaches exhibited highly consistent trends in changes in oxidative stress markers (SOD, MDA, and ROS) and ferroptosis biomarkers (ferrous ions, GSH, and GPX4).

Notably, we validated the changes in apoptosis-related proteins both *in vitro* and *in vivo*. The increased Bcl-2/Bax ratio and decreased Cleaved-Caspase-3/Caspase-3 ratio confirm the role of BGBP in apoptosis inhibition. This finding is consistent with that of Zhang, who demonstrated that electroacupuncture pretreatment mitigated cerebral ischemia-reperfusion injury through similar mechanisms (39). These findings suggest that BGBP exerts its neuroprotective effects by synergistically inhibiting multiple cell death pathways, including ferroptosis and apoptosis.

This consistency between the *in vivo* and *in vitro* results not only enhances the credibility of our conclusions but also indicates that BGBP has a clear translational medical value in SCIRI therapy.

The innovation of this study is primarily reflected in the following aspects. It is the first to explore the protective role and mechanism of BGBP in SCIRI, providing new evidence for the development of medicinal value in traditional edible plants. This study explicitly proposes a mechanism by which BGBP regulates neuronal ferroptosis through the Nrf2/HO-1 pathway, thereby enriching the pathophysiological theories of SCIRI. By combining *in vivo* and *in vitro* experiments, this study constructed a comprehensive evidence chain from the overall behavior to molecular events, enhancing the reliability of the conclusions. Beyond focusing solely on ferroptosis, this study also concurrently analyzed other related cell death pathways, such as apoptosis, providing a scientific rationale for multitargeted therapies.

From a translational medicine perspective, this study identified a potential natural drug candidate for the prevention and treatment of SCIRI. As a widely consumed plant, bitter gourd has established safety profiles, providing favorable conditions for its subsequent development. Furthermore, an *in vivo* dose of 50 mg/kg and *in vitro* concentration of 1.6 mg/mL we served as critical reference parameters for future studies.

Although this study yields meaningful findings, several limitations must be addressed in future work. First, although BGBP activated the Nrf2 pathway, the necessity of this pathway was not confirmed using specific Nrf2 inhibitors (e.g., ML385) or genetic knockout models.

Second, transcriptomic and metabolomic analyses of the relevant tissues were not performed to identify other potential targets. Future sequencing studies on these tissues could reveal potential therapeutic targets. Furthermore, BGBP concentrations were not measured in plasma or spinal cord tissue in this study; therefore, actual exposure levels at the target site remain unknown. The gastrointestinal stability and oral bioavailability of BGBP also remain to be characterized, which is a common limitation in peptide-based nutritional intervention studies (40). The pharmacokinetic characteristics of BGBP remain unclear, and future research should focus on its bioavailability. However, the role of inflammatory cascades in SCIRI remains unclear. Future studies should investigate the inflammation-related pathways and their therapeutic targets. The current focus is primarily on the nervous system itself, with insufficient exploration of its effects on other systems (e.g., cardiovascular and digestive systems). Previous studies have indicated that the Nrf2/HO-1 pathway also plays a significant role in these systems (41).

Future research should explore the synergistic effects of BGBP and other neuroprotective agents, investigate the efficacy of BGBP in chronic spinal cord injury or aged animal models (12), and examine the specific target sites of BGBP within the iron metabolism regulatory network.

## 5 Conclusion

In summary, this study provides the first evidence that BGBP protects against SCIRI by activating the Nrf2/HO-1 pathway, thereby alleviating oxidative stress and inhibiting neuronal ferroptosis. Methodologically, the combination of the CoCl_2_-induced BV-2 cell model and the refined 90-minute rat abdominal aortic clamping model offers a practical and reproducible platform for studying ferroptosis-targeted neuroprotection. From a translational perspective, BGBP—a food-derived peptide with an established safety profile—represents a promising candidate for preoperative nutritional strategies in patients at risk for spinal cord ischemia.

## Data Availability

The original contributions presented in the study are included in the article/Supplementary material, further inquiries can be directed to the corresponding author.
