# Supplementary material for: Bitter gourd bioactive peptide alleviates neuronal ferroptosis after spinal cord ischemia-reperfusion injury, combined with emerging cell and animal models
Source: Front Nutr. 2026 May 11;13:1850363. doi: 10.3389/fnut.2026.1850363 (PMC13199010; doi:10.3389/fnut.2026.1850363)
Supplement: Supplementary file 1 [file Table_1.docx]

Bitter gourd bioactive peptide alleviate neuronal ferroptosis after spinal cord ischemia-reperfusion injury, combined with emerging cell and animal models

Qiyang Diao^a †^, Ming Nuo^b†^, Qimuge Suyila^c^ , Yongzhen Nie*, Xiulan Su*

^a^ Department of Anesthesiology,Peking University Cancer Hospital (Inner Mongolia Campus)/Affiliated Cancer Hospital of Inner Mongolia Medical University, Hohhot, 010020, China

^b^ Department of Anesthesiology, Inner Mongolia Autonomous Region People’s Hospital, Hohhot, 010017, China

^c^ Clinical Medical Research Center of the Affiliated Hospital, Inner Mongolia Medical University, Hohhot, 010050, China

^†^ These authors contributed equally to this work

* Corresponding author:

Xiulan Su, Clinical Medical Research Center of Affiliated Hospital, Inner Mongolia Medical University, Inner Mongolia Bioactive Peptide Engineering Laboratory, Hohhot, 010050, China; E-mail: [xlsu11@nmgfy.com](mailto:xlsu@nmgfy.com)

Yongzhen Nie, Department of Anesthesiology,Peking University Cancer Hospital (Inner Mongolia Campus)/Affiliated Cancer Hospital of Inner Mongolia Medical University, Hohhot, 010020, China;E-mail:nyz1@163.com

**Materials and Methods**

**Animal Model and Behavioral Assessment**

| ****Chart 1: Tarlov Scale for Hindlimb Motor Function Assessment in Rats**** | |
| --- | --- |
| Score | Description |
| 0 | No lower extremity movement |
| 1 | Perceptible joint movement without gravity |
| 2 | Active movement but unable to stand against gravity |
| 3 | Able to stand and walk with obvious deficit |
| 4 | Normal gait, no observable deficit |

Adapted from standard neurological assessment protocols for rodent models of spinal cord injury.

| ****Chart 2: Basso, Beattie, Bresnahan (BBB) Locomotor Rating Scale (Simplified Key Points)**** | |
| --- | --- |
| Score Range | ****General Functional Description**** |
| 0 | No observable hindlimb movement |
| 1-7 | Isolated joint movements → extensive joint movement without weight support |
| 8-12 | Plantar placement without weight support → frequent weight-supported stepping |
| 13-16 | Consistent weight-supported stepping, consistent coordination, and predominant parallel paw position |
| 17-21 | Consistent coordinated gait, trunk stability, tail position, and fine paw placement |

*Note: The BBB scale is a 22-point (0-21) detailed scale. The above table summarizes the primary functional milestones. The full scale assesses finer details of locomotion, including joint movement, paw placement, trunk stability, and tail position.

****Videos S1-S4.** **Inclined Plane Test and Simple Open Field Test****

****Simple Open Field Test (Videos S1-S3):****
This assay assesses general locomotion and exploratory behaviour in a flat, enclosed arena. Rats with paraplegia exhibit **reduced total movement distance and limited exploration** (especially in the central area), demonstrating deficits in spontaneous motor activity and increased anxiety-like behaviour.

****Inclined Plane Test (Video S4):****
This test evaluates limb strength and balance by placing the rat on an adjustable inclined board. Paralysed rats show a **significant decrease in the maximum angle they can maintain**, reflecting impaired motor function and poor grip strength due to hindlimb paralysis.

**Cell Incucyte**

****Videos S5-S8.Real-time Live-cell Analysis (24 hours)****

Cell proliferation and morphology were dynamically monitored for 24 hours using the Cell Incucyte® Live-Cell Analysis System (Essen BioScience). BV-2 cells were seeded in a 96-well plate and treated according to the experimental groups (Ctrl(Video S5), CoCl₂(Video S6), Ctrl+BGBP(Video S7), CoCl₂+BGBP(Video S8)). The system acquired phase-contrast images from each well every 4 hours. Cell confluence or count was quantified automatically for the entire duration to generate growth curves.
